# Cholangiocarcinoma in a Resected Biliary Cyst: Importance of Follow-up

**DOI:** 10.7759/cureus.4532

**Published:** 2019-04-24

**Authors:** Mustafa N Malik, Tabinda Saleem, Shehroz Aslam, Rida Riaz, Muhammad Abdullah Yousaf

**Affiliations:** 1 Internal Medicine, The University of Arizona, Tucson, USA; 2 Internal Medicine, Shifa International Hospital, Islamabad, PAK; 3 Internal Medicine, Maricopa Medical Center, Phoenix, USA; 4 Internal Medicine, Nawaz Sharif Medical College - University of Gujrat, Gujrat, PAK; 5 Internal Medicine, Rawalpindi Medical University, Islamabad, PAK

**Keywords:** biliary cyst, choledochal cyst, cholangiocarcinoma, adenocarcinoma

## Abstract

Biliary cysts are rare cystic dilatations of the biliary tree. Biliary cysts are positively associated with several significant complications, amongst them, cholangiocarcinoma befalls the most dreadful one. The elevated incidence is 20-30% in the unresected cyst and 0.7% in resected cysts. Magnetic resonance imaging (MRI) scan, magnetic resonance cholangiopancreatography (MRCP) or a contrast-enhanced computed tomography (CECT) is applied for the initial diagnostic study but the ultimate diagnosis ordinarily requires the tissue biopsy. Currently, the sole curative option involves the complete surgical resection of the lesion, with standard chemotherapy and active radiation applied as an alternative for the unresectable tumors. Despite the curative surgery the percentage of eternal recurrence of the tumor indefinitely persists, and effective post-surgical surveillance is reasonably demanded. We report a case of 29-year-old female with local recurrence of cholangiocarcinoma in a previously resected biliary cyst type I. The curative resection of the choledochal cyst only minimizes the considerable risk of the possible development of future cholangiocarcinoma but it does not completely prevent it. The appropriate follow-up for potential patients who have been typically treated for a biliary cyst is unclear. The lethal course of cholangiocarcinoma is believed due to its slow asymptomatic growing phase. Therefore, to adequately screen for malignancy, periodic imaging along with annual liver tests represents a reasonable approach to prevent the possible development of this appalling complication.

## Introduction

Biliary cysts, formerly known as the choledochal cysts, are rare cystic dilatations of the biliary tree with an elevated incidence of 1:100,000. The likely female to male ratio is invariably 4:1 with a marked predilection for Asian ethnicity [1]. Biliary cysts can be congenital or acquired and are associated with various anatomic abnormalities. Among its various complications, cholangiocarcinoma is undoubtedly the most dreadful one with a 20-to-30-fold increased risk of malignancy in biliary cysts compared with the general population. It is typically associated with all subtypes of the biliary cyst but is probably most common in specific type IV cysts and I. Early complete surgical resection of the cyst severely reduces the elevated incidence of cholangiocarcinoma to 0.7% but the slight risk remains in previously or partially resected cysts [2]. Latterly, the term cholangiocarcinoma is applied to precisely represent bile duct cancers that invariably arise from the epithelial cells of the intrahepatic, perihilar or extra-hepatic (distal) biliary tree, exclusive of the gall bladder and the ampulla of Vater. The rare cancer strikingly includes a variable presentation from an asymptomatic lesion to life-endangering widespread metastatic disease. Despite the complete surgical resection of the tumor the chance of eternal recurrence of cancer indefinitely persists, and effective post-surgical surveillance is reasonably demanded. We report a case of local recurrence of cholangiocarcinoma in a previously resected biliary cyst type I.

## Case presentation

A 29-year-old female of Asian ethnicity presented to the medical outpatient department with chief complaints of progressive jaundice and continuous dull pain in the right hypochondrium from the past seven months. She also reported an unintentional weight loss of 22 lbs during the corresponding period. On physical examination, she was severely icteric and cachectic with an abdominal examination revealing tender hepatomegaly and splenomegaly. On chest auscultation, she invariably had bilateral coarse basal crepts. Blood investigations precisely revealed an obstructive pattern of liver enzymes with raised serum bilirubin of 21.9 mg/dl with direct 20.0 mg/dl, alkaline phosphatase (ALP) 1854 U/L and gamma-glutamyl transferase (GGT) of 2022 U/L with markedly deranged prothrombin time (PT) and activated partial thromboplastin time (APTT). Aspartate aminotransferase (AST) and alanine aminotransferase (ALT) and all other routine investigations were normal. Ultrasonography (USG) of the abdomen showed an enlarged liver with focal lesions with no hepatic biliary dilatation.

**Figure 1:**
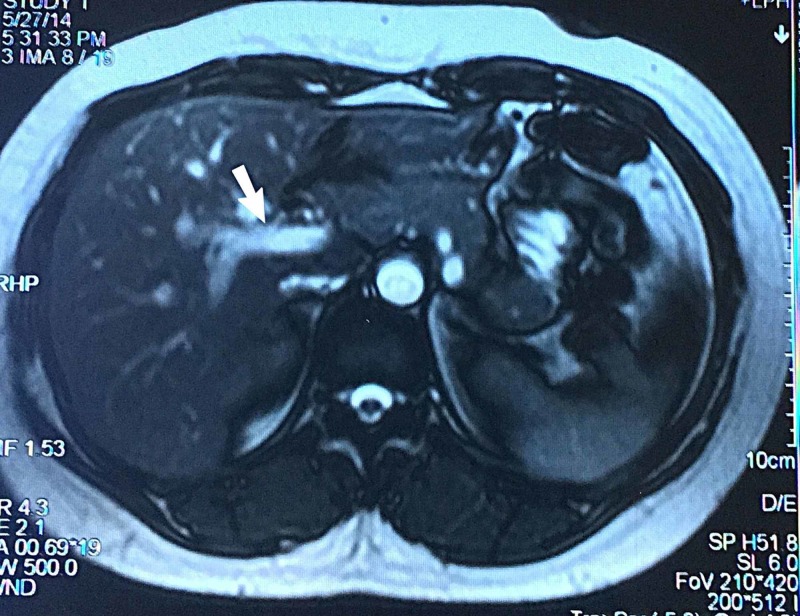
Magnetic resonance cholangiopancreatography (MRCP) showing fusiform dilatation of common bile duct measuring 2.3 cm in transverse dimension along with dilatation of common hepatic duct suggestive of type I biliary cyst.

**Figure 2:**
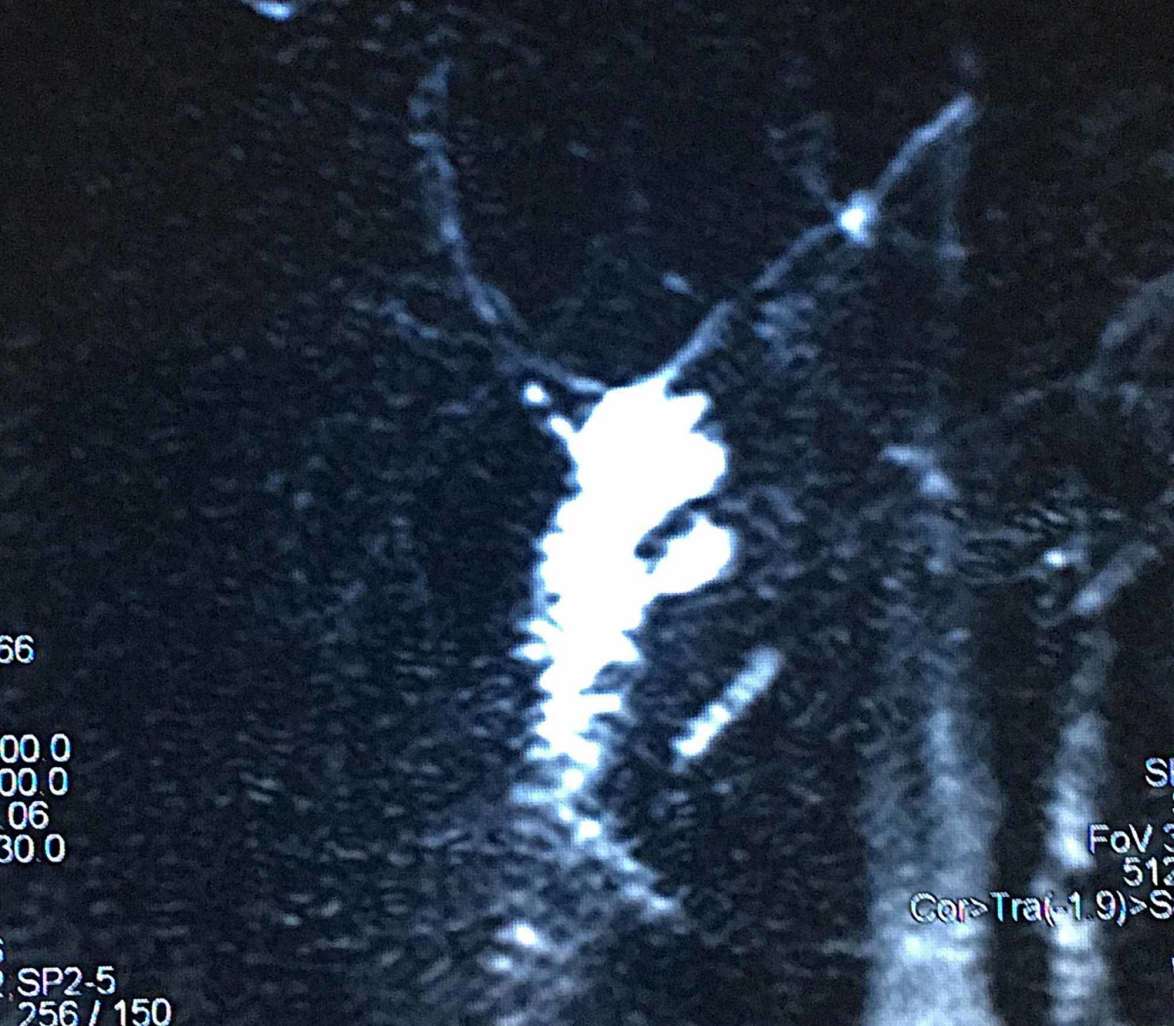
Magnetic resonance cholangiopancreatography (MRCP) image showing type I biliary cyst.

Previous surgical history was significant for biliary cyst diagnosed four years back. Magnetic resonance cholangiopancreatography (MRCP) report at that specific time showed a 2.3 cm fusiform dilatation of common bile duct, positively confirming the potential diagnosis of biliary cyst type I (Figures 1, 2).

She underwent complete cyst resection and cholecystectomy with Roux-en-Y hepaticojejunostomy. A biopsy at that particular point showed no signs of malignancy. Her immediate postoperative course was unremarkable and no post-operative surveillance was routinely done.

**Figure 3:**
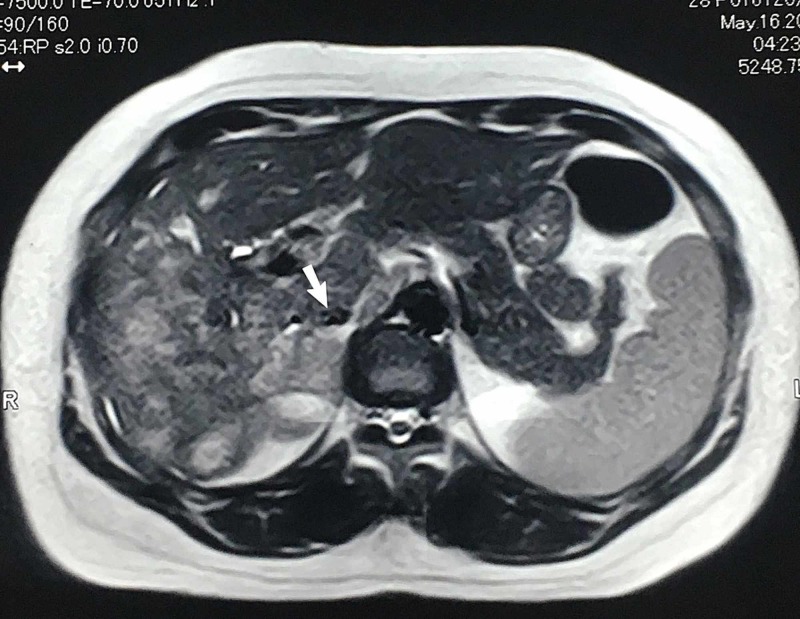
Magnetic resonance cholangiopancreatography (MRCP) findings suggestive of ill-defined heterogeneously T2WS hyperintense signal intensity area in right hepatic lobe reaching up to porta hepatis causing obliteration of common hepatic duct with evidence of mild prominence of intrahepatic biliary channels.

**Figure 4:**
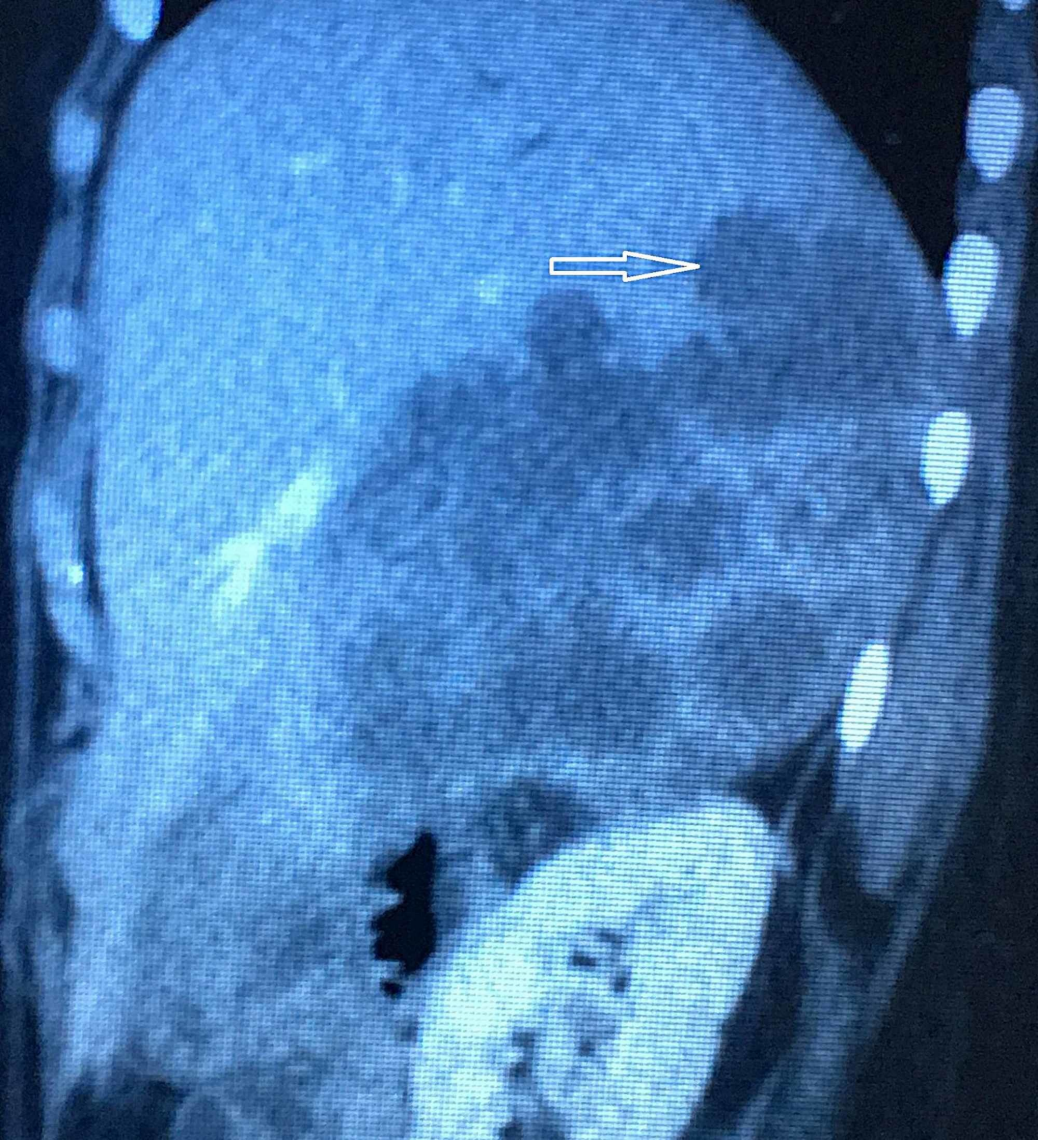
Contrast-enhanced computed tomography (CECT) scan of the abdomen showing multiple ill-defined low-density areas in the right lobe of the liver replacing the liver parenchyma, the largest measuring 90 x 78 mm.

**Figure 5:**
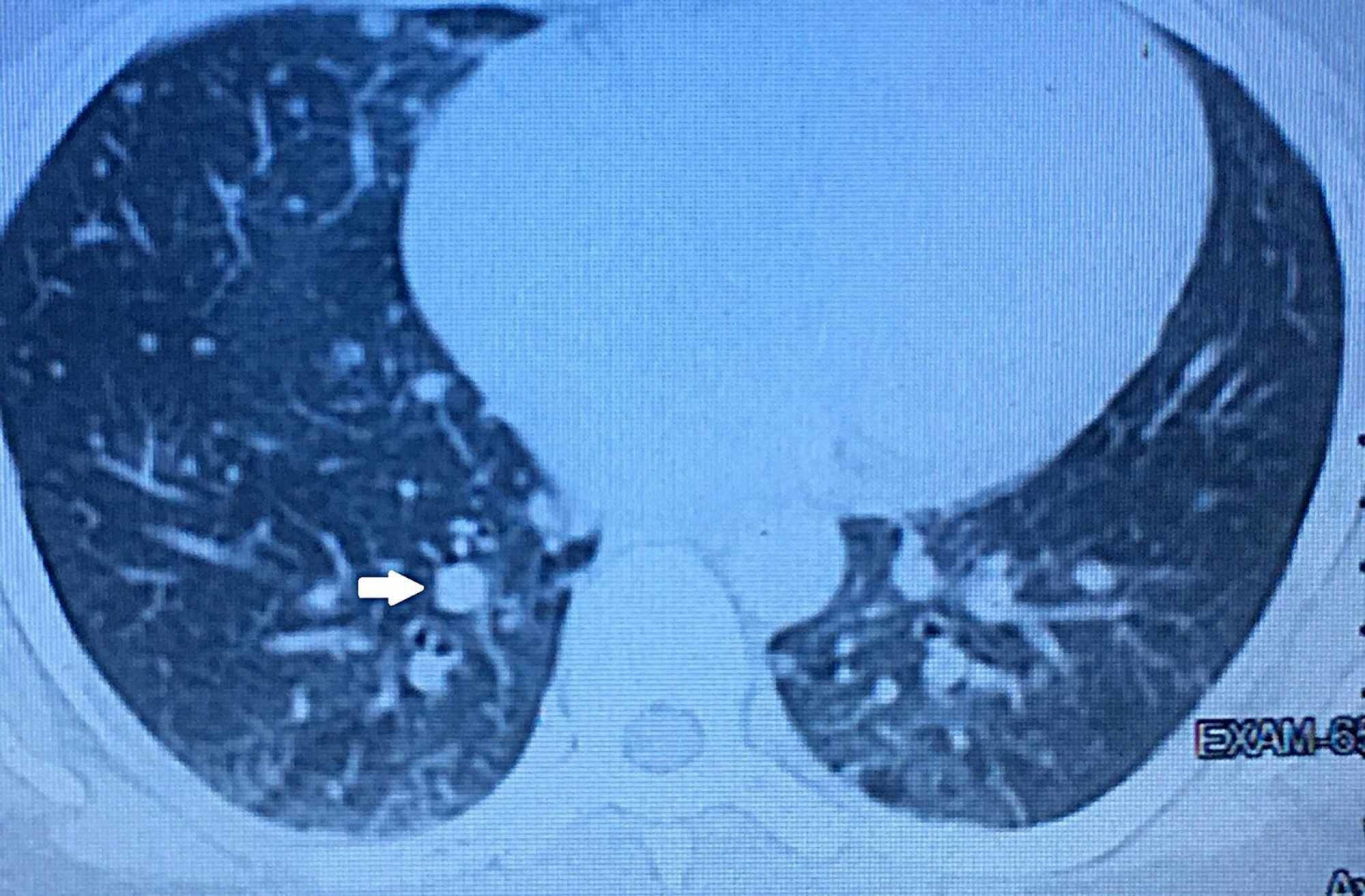
Contrast-enhanced computed tomography (CECT) lung window showing multiple nodular densities of variable sizes scattered bilaterally in the lung parenchyma, the largest measuring 11 x 8.7 mm.

A contrast-enhanced computed tomography scan (CECT) and magnetic resonance cholangiopancreatography were planned. They both suggested multiple masses in the liver that were obstructing the common hepatic duct resulting in jaundice with metastatic spread to regional lymph nodes and lungs (Figures 3-5).

For the histopathological confirmation of the cancer type, image-guided fine needle aspiration cytology (FNAC) was performed adequately. The biopsy sample sufficiently revealed malignant neoplastic lesions with prominent glandular differentiation positive for cytokeratin 7, 19 and CDX-2 suggesting metastatic adenocarcinoma most likely cholangiocarcinoma.

Liver resection was not possible due to the widespread metastatic disease at official presentation, and she was cautiously started on intensive chemotherapy. Her condition deteriorated after her fourth chemotherapy session, resulting in multi-organ failure. Palliative care was adequately provided but she expired within a few abysmal weeks due to the potential complications of organ failure.

## Discussion

Biliary cysts are rare cystic dilatations of the biliary tree that may occur singly or in multiples throughout the intra-hepatic or extra-hepatic biliary channels. Formerly the biliary cysts were popularly known as the choledochal cyst due to their possible involvement of the extra-hepatic bile ducts. However, the standard classification was carefully revised in 1977 by Todani et al. to appropriately include the intrahepatic cysts [3]. This classification was further revised in 2003 to incorporate the abnormal pancreaticobiliary junction (APBJ) [4]. The current classification precisely defines six unique types of biliary cysts (Table 1) [5, 6].

**Table 1:** Types of biliary cysts as per the revised classification.

Types of Biliary Cysts	Prevalence	Brief Explanation [5, 6]
Type I	50-85%	Dilatation of the extra-hepatic common bile duct. Associated with abnormal pancreaticobiliary junction (APBJ).
Type II	2%	True diverticulum from extra-hepatic bile ducts.
Type III	1-5%	Dilatation of extra-hepatic bile duct within the duodenal wall (choledochocele).
Type IV	15-35%	Cysts involving both intra- and extra-hepatic ducts.
Type V	20%	Multiple dilatations or cysts of intra-hepatic bile ducts only (Caroli disease).
Type VI	Rare	Isolated cystic dilatations of the cystic duct.

The characteristic presentation of a biliary cyst traditionally includes an abdominal mass that presents with abdominal pain and jaundice, but only a few patients present with the classic symptoms [7]. Most patients present with the vague symptoms of abdominal discomfort that is frequently confused for gastroesophageal reflux disease (GERD) or cholelithiasis. The most effective way to sufficiently establish the accurate diagnosis of a biliary cyst is conveniently through USG and CECT/MRCP [8]. On USG, a biliary cyst typically appears as a water-density mass at the porta hepatis or adjacent to the head of the pancreas, with varying degrees of intrahepatic biliary dilatation. Whereas on CECT/MRCP, it appears as a well-demarcated water attenuation lesion with no contrast enhancement. Due to the significant risk of possible complications, complete surgical resection is unanimously recommended with cholecystectomy along with Roux-en-Y hepaticojejunostomy being precisely the standard procedure of choice [9].

Possible complications of biliary cysts principally result from stasis and typically include cholangitis, choledocholithiasis, recurrent pancreatitis, cirrhosis, and portal hypertension [10]. However, the most lethal complication among all is undoubtedly cholangiocarcinoma which results from chronic irritation and local inflammation of the mucosa. It typically has a rare incidence of 10-30%, and the risk is indeed more significant after the drainage procedures. It naturally arises from the local cells within the bile ducts (both intra- and extrahepatic) and typically tends to grow gradually. After infiltrating the impregnable walls of the bile ducts, it dissects into tissue planes with rapid local extension into the liver, porta hepatis, and regional lymph nodes. It traditionally presents with the classic symptoms of abdominal pain, obstructive jaundice, mild fever, and significant weight loss. USG, CT scan or MRCP helps in aiding the accurate diagnosis but tissue biopsy is typically required for the ultimate confirmation. Currently, the sole curative option undoubtedly includes the complete surgical resection of the discovered tumor and bile duct along with a significant portion of the liver with Roux-en-Y hepaticojejunostomy. The standard chemotherapy and active radiation are frequently used for non-resectable tumors. Cholangiocarcinoma typically developing in a biliary cyst especially the type IV and I, invariably has early morbidity. The dismal prognosis of the likely cancer is undoubtedly owing to delayed diagnosis with a relatively rapid metastatic course. Lymph node involvement and possible depth of tumor invasion are important prognostic indicators as reflected in the tumor node and metastasis (TNM) staging criteria. This criterion was currently revised by the combined American Joint Committee on Cancer (AJCC) or the Union for International Cancer Control (UICC). These changes have led to better prognostic stratifications [11, 12]. Five-year survival rates range from 20-50 percent but are as high as 54-62 percent in selected patients, who undergo complete resection of a node-negative tumor [13]. However, for certain patients with lymph node-positive disease, five-year survival rates are woefully under 20 percent [14]. Prompt initiation of intensive chemotherapy can carefully limit the gradual spread and can appreciably increase the life expectancy in some fortunate patients.

While the carcinoma risk decreases with prior resection of the cyst, these selected patients continue to be at considerable risk. The elevated incidence of progressively developing cholangiocarcinoma in the remaining biliary tree compared to the general population remains high in these selected patients. Post-excisional malignant disease is typically seen in 0.7 to six percent of compelling cases [2]. This can arise either from the anastomotic site, the remnant of cystic tissue or the subclinical malignant disease at the apparent time of curative resection. Despite this, the appropriate follow-up for potential patients who have been typically treated for a biliary cyst is unclear.

## Conclusions

We reasonably conclude that along with prompt diagnosis and complete resection of a biliary cyst, regular follow-up is necessary to prevent the gradual development of cholangiocarcinoma. Successful surgery adequately provides a unique possibility for the cure. However, only a minority of patients presents with an early stage disease and are independent candidates for curative resection. Furthermore, the likely outcomes after curative resection are unsatisfactory, particularly with node-positive disease. Annual imaging with serial MRI, MRCP, CECT or intraductal ultrasound along with successive liver function tests, can be helpful to identify malignant changes at a primitive stage in these susceptible patients.
